# The effects of standard and modified LSVT BIG therapy protocols on balance and gait in Parkinson's disease: A randomized controlled trial

**DOI:** 10.1002/brb3.3458

**Published:** 2024-03-07

**Authors:** Sefa Eldemir, Kader Eldemir, Fettah Saygili, Cagla Ozkul, Rezzak Yilmaz, Muhittin Cenk Akbostancı, Arzu Guclu‐Gunduz

**Affiliations:** ^1^ Department of Physiotherapy and Rehabilitation, Faculty of Health Sciences Sivas Cumhuriyet University Sivas Türkiye; ^2^ Department of Physiotherapy and Rehabilitation, Faculty of Health Sciences Ordu University Ordu Türkiye; ^3^ Department of Physiotherapy and Rehabilitation, Faculty of Health Sciences Aydın Adnan Menderes University Aydın Türkiye; ^4^ Department of Physiotherapy and Rehabilitation, Faculty of Health Sciences Gazi University Ankara Türkiye; ^5^ Department of Neurology and Ankara University Brain Research Center Ankara University, School of Medicine Ankara Türkiye

**Keywords:** balance, LSVT BIG, Parkinson's disease, physiotherapy

## Abstract

**Background:**

Lee Silverman voice treatment (LSVT) BIG is an exercise program developed for patients with Parkinson's disease (PwPD), consisting of sets of exercises performed for 4 consecutive days a week for 4 weeks. However, the standard protocol suggests a treatment frequency difficult to follow for most patients who have difficulties reaching rehabilitation clinics. Our aim was to compare the standard LSVT BIG protocol with a modified LSVT (m‐LSVT) BIG protocol (twice a week in the clinic for 4 weeks and twice a week at home for 4 weeks).

**Methods:**

In this randomized controlled trial, 16 PwPD (aged 18–80 years, Hoehn and Yahr stages I–III) were recruited into two groups. The LSVT group received standard LSVT BIG training (four times per week for 4 weeks at the clinic). The other group was given m‐LSVT BIG exercises, but unlike the LSVT group, the m‐LSVT group exercised twice a week at the clinic and twice a week at home for 4 weeks. The Berg Balance Scale was used to assess functional balance. Biodex Balance System was used to assess laboratory balance measures. Timed Up and Go test and G‐Walk sensor system were used to assess functional mobility and spatiotemporal gait analysis.

**Results:**

Significant group‐by‐time interactions on the eyes open‐firm surface score of the modified clinical test of sensory integration of balance (*F* = 10.138, *p* = .007) and gait cycle symmetry index (*F* = 10.470, *p* = .010) were found to be in favor of the LSVT group. Additionally, post hoc analyses revealed that both groups significantly improved postural stability, gait speed, motor symptoms, and functional mobility (*p* < .05).

**Conclusion:**

The results revealed the beneficial effects of the modified protocol on balance and gait in PwPD, as well as the superiority of the standard LSVT BIG protocol. The m‐LSVT BIG protocol may be an effective intervention method, especially for PwPD who have difficulty adapting to the treatment frequency of the standard protocol.

## 1 INTRODUCTION

Current basics of Parkinson's disease (PD) treatment include medications (levodopa is considered the “gold standard”) and surgical approaches (Rascol et al., 2011). However, even with optimal medical or surgical treatment, patients with PD (PwPD) experience progressive non‐dopa‐responsive problems such as impairment in gait and balance. These affect the activities of daily living (ADLs) and the quality of life (QoL) of patients negatively over the years (Debû et al., 2018).

Physical exercise is applied as an adjunct to pharmacological and surgical treatments to maximize functional capacity, improve QoL, and minimize complications (Abbruzzese et al., 2016). The effects of exercise on neural plasticity have been explored (Francardo et al., 2017; Will et al., 2004). Accordingly, important principles have been identified, such as the intensity, specificity, difficulty, and complexity of the practice, which could potentially have a lasting impact on neuroplasticity and behavior (Petzinger et al., 2010).

Recently, a technique named “LSVT BIG” derived from Lee Silverman Voice Therapy (LSVT LOUD) has been shown to improve motor performance (Ebersbach et al., 2010, 2015; Farley et al., 2008; Fırat et al., 2023), gait (Ebersbach et al., 2015; Farley & Koshland, 2005), and reach (Farley & Koshland, 2005), ADL (Fırat et al., 2023), and QoL (Fırat et al., 2023) in PD. LSVT BIG training aims to recalibrate the sensory perception of the normal amplitude of movements while focusing on high‐amplitude movements with multiple repetitions, high intensity, and increasing complexity. In addition, LSVT BIG training encourages the patients to use their gains in their daily lives by expanding the exercises out of the clinical setting (Fox et al., 2012). For example, in order to preserve the improvement in walking, the patient is taught to pay attention to his gait while coming to the clinic or going to work. Thanks to these features, LSVT BIG training is unique in that it provides both intensive training and the opportunity to transfer improvements to daily life (Fox et al., 2012).

Although training intensity is crucial to the success of LSVT BIG, the standard protocol applied 4 consecutive days a week (16 sessions, 4 weeks) may strain patients in terms of time and economy, and thus adherence to outpatient treatment may decrease (Ebersbach et al., 2015; Schootemeijer et al., 2020). Therefore, the aim of the current study was to compare the LSVT BIG standard protocol with a modified LSVT (m‐LSVT) BIG protocol (twice a week for 4 weeks at the clinic and twice a week for 4 weeks at home).

## 2 METHODS

### 2.1 Participants

PwPD were referred from the Movement Disorders Unit at Ankara University School of Medicine Department of Neurology. Outcome measures and exercise interventions were performed at Gazi University, Department of Physiotherapy and Rehabilitation, neurorehabilitation outpatient clinic. PwPD were diagnosed according to the UK Brain Bank Criteria. Patients with Hoehn and Yahr (H&Y) stages I–III, aged 18–80 years, with no dementia (a Mini‐Mental State Examination score of at least 24) were included. Individuals with additional neurological disorders, disabling dyskinesias, or any musculoskeletal disorder in which exercise was contraindicated were excluded.

The study was approved by the local ethics committee and was registered at ClinicalTrials.gov (ClinicalTrials.gov ID: NCT05520541) before the recruitment started. All procedures were in accordance with the Declaration of Helsinki, and written informed consent was obtained from all participants.

### 2.2 Study design

**FIGURE 1:**
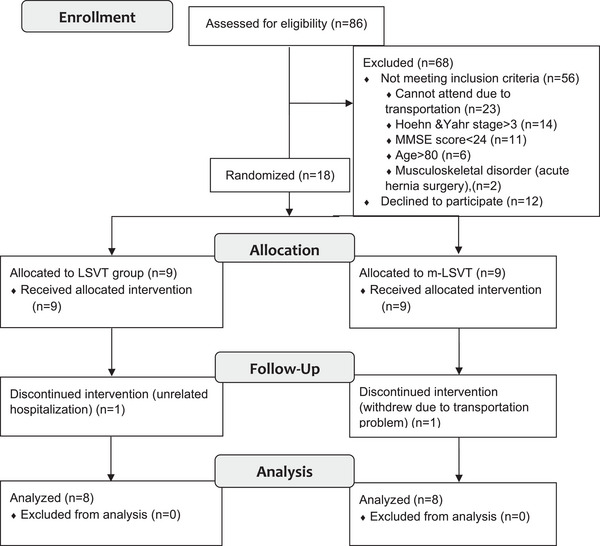
Participants flow through the study. Consolidated standards of reporting trials (CONSORT) flow chart.

The current study was designed as a randomized, controlled, single‐blind trial. Individuals were randomly assigned to the standard LSVT BIG (LSVT group) protocol and the modified LSVT BIG (m‐LSVT group) protocol using a minimization method. Minimization is a randomization method that aims to produce the best overall balance between treatment groups according to patient characteristics (Taves, 2010). MinimPy, a minimization program, was used to balance the gender and clinical course of participants (Saghaei, 2011). Two physiotherapists blinded to patient groups assessed all participants at baseline and after training. The study design and participants are shown in Figure 1.

### 2.3 Interventions

One physiotherapist (S.E.) certified as the LSVT BIG instructor delivered all exercise sessions. Patients assigned to the LSVT group received the standard LSVT BIG protocol of 16 1‐h sessions (four times per week for 4 weeks at the clinic) one‐to‐one at the clinic. Patients assigned to the m‐LSVT group received 8 1‐h sessions (twice a week for 4 weeks at the clinic) one‐to‐one at the clinic and 8 1‐h sessions (twice a week for 4 weeks at the home) unsupervised at home.

LSVT BIG training has previously been described in detail (Farley et al., 2008). Briefly, half of the exercises consist of standardized repetitive multidirectional movements with maximal amplitude (e.g., stepping and reaching) and stretches (i.e., reaching and turning sideways while sitting and standing). The second half of exercise includes task‐oriented ADLs according to individual needs and preferences (i.e., buttoning a shirt and donning a jacket). Throughout their program, individuals were instructed to perform at 80% of their maximum perceived effort, verbally encouraging sentences were used to target intense motivation, and feedback was provided during all exercises by the instructor. Additionally, individuals were taught to use bigger movements to provide continuous exercise in everyday activities. In all sessions in the clinic, their spouses or caregivers were asked to participate in the exercises from the first session, and in the first session, functional goals were determined together, and a detailed exercise program was created. Patients were asked to be accompanied by their spouses or caregivers to avoid injury during exercises at home. In addition, to perform the exercises correctly and safely, a detailed video recording of the session was sent to the patients by the therapist, and a phone number that they could reach at any time was given.

### 2.4 Assessment procedures

Participants were assessed before starting active treatment (pre) and after completing treatment (post). Demographic variables (age, gender, and body mass index [BMI]) were recorded before starting active treatment. The Turkish version of the Unified Parkinson's Disease Rating Scale‐Motor score (UPDRS‐III) was used to assess the severity of motor symptoms (Akbostancı et al., 2000).

Berg Balance Scale (BBS) was used to assess functional balance (Sahin et al., 2008). Biodex Balance System (Biodex, Inc.) was used to assess laboratory balance measures consisting of the postural stability (PS) test, limit of stability (LOS) test, and the modified clinical test of sensory integration of balance (m‐CTSIB) (Cachupe et al., 2001). m‐CTSIB assesses the sensory integration when one or more senses are compromised, and patients perform this in four conditions; (1) open eyes‐firm surface, (2) closed eyes‐firm surface, (3) open eyes‐foam surface, and (4) closed eyes‐foam surface. Timed Up and Go (TUG) test (Mollinedo & Cancela, 2020) and G‐Walk sensor system (BTS‐Bioengineering Company) (De Ridder et al., 2019) were used to assess functional mobility and spatiotemporal gait analysis, respectively.

### 2.5 Statistics

The sample size calculation was based on the significant improvement of the TUG test score observed in a similar intervention study in PD (Ebersbach et al., 2015). Their findings provided a Cohen's *d* effect size of 0.844. To achieve 80% power with a two‐sided level of 5%, the sample size required per group was 7 using G*Power analysis software (version 3.1; Heinrich Heine Universitaet) (Faul et al., 2007). Assuming a 15% dropout rate, we recruited nine participants per group.

The Shapiro–Wilk test was used to test for the normality of data. Demographic characteristics of participants were compared among the groups using the Mann–Whitney *U* test (BMI, disease duration), the independent sample's *t*‐test (age) according to the normality of data, or the chi‐square test (gender, dominant side, and H&Y stage).

For normally distributed outcome variables, the 2 × 2 factorial analysis of variance was used with time (pre vs. post) and group (LSVT group vs. m‐LSVT group) as the independent variables for the outcome variables. Post hoc comparisons were assessed using the Bonferroni corrections. For non‐normally distributed outcome variables, the Mann–Whitney *U* test was used to determine between‐group differences. The Wilcoxon signed rank test was used to determine within‐group differences. Normally distributed outcome variables were presented as mean ± standard deviation (SD), and non‐normally distributed outcome variables were presented as median (interquartile range). Analyses of the outcome measurements were done per protocol. The significance level was set at *p* = .05. The SPSS software (version 22.0; SPSS, Inc.) was used for the statistical analysis.

## 3 RESULTS

A flow chart of the study is presented in Figure 1. Out of 86 PwPD assessed for eligibility, 16 were included. The demographic variables, disease duration, and PD stage were similar among the groups (*p* > .05) (Table 1). In addition, there was no difference between the outcome measures of the groups in the pretest (*p* > .05).

**TABLE 1:** Demographic characteristics of participants.

	LSVT (*n* = 8)	m‐LSVT (*n* = 8)	*p*
**Age (years)**	62.50 ± 8.53	69.50 ± 10.58	.167
**BMI (kg/m^2^)**	26.33 (24.16–28.83)	27.10 (26.59–31.07)	.293
**Gender, female/male (female %)**	2/6 (25%)	1/7 (12.5%)	.522
**Dominant side, right/left (right %)**	8/0 (100%)	7/1 (87.5%)	.302
**Disease duration (months)**	30.0 (24.0–76.50)	60.0 (31.5–96.0)	.392
**Hoehn and Yahr stage, *n* (%)**			
1	3 (37.5%)	3 (37.5%)	
2	2 (25%)	3 (37.5%)	.819
3	3 (37.5%)	2 (25%)	

*Note*: Values are mean ± SD, median (IQR), or as otherwise indicated, *p* < .05.

Abbreviations: BMI, body mass index; LSVT, Lee Silverman voice treatment; m‐LSVT, modified LSVT.

Values of PS, LOS, m‐CTSIB, and spatiotemporal gait analysis are reported in Table 2. There were significant interaction effects (time × group) for eyes open‐firm surface score of m‐CTSIB and gait cycle symmetry index (*F* = 10.138, *p* = .007, and *F* = 10.470, *p* = .010, respectively) (Table 2). Post hoc analyses revealed that both groups significantly improved PS and gait speed (*p* < .05). Additionally, the LSVT group showed significant improvements in the limits of stability, m‐CTSIB scores excluding eyes open‐foam surface score, cadence, nondominant side step length, swing phase, and gait cycle symmetry index (*p* < .05).

**TABLE 2:** Treatment effects on laboratory balance and spatiotemporal gait analysis for the Lee Silverman voice treatment (LSVT) group and modified LSVT (m‐LSVT) group at baseline and 4‐week follow‐up.

		Pretest mean ± SD	Posttest mean ± SD	Mean difference (95% CI)	*p* (within group)	Interaction (time × group)		
						*F*	*p*	*ηp* ^2^
**Balance scores**								
**PS**	LSVT m‐LSVT	0.52 ± 0.26 0.45 ± 0.42	0.37 ± 0.16 0.35 ± 0.28	−0.15 (−0.06 to (−0.24)) −0.10 (−0.01 to (−0.19))	.003* .033*	.700	.417	0.048
**LOS**	LSVT m‐LSVT	49.62 ± 16.09 54.62 ± 16.80	64.50 ± 8.99 62.50 ± 12.19	14.87 (6.34–23.41) 7.87 (−66.0 to 16.41)	.002* .068	1.547	.234	0.100
**m‐CTSIB** Eyes open‐firm surface Eyes closed‐firm surface Eyes open‐foam surface Eyes closed‐foam surface	LSVT m‐LSVT LSVT m‐LSVT LSVT m‐LSVT LSVT m‐LSVT	0.70 ± 0.30 0.58 ± 0.32 1.17 ± 0.12 1.04 ± 0.55 1.23 ± 0.74 0.87 ± 0.30 2.81 ± 1.31 2.42 ± 1.10	0.53 ± 0.32 0.59 ± 0.30 0.85 ± 0.50 0.86 ± 0.52 0.92 ± 0.50 0.81 ± 0.36 2.06 ± 1.01 1.96 ± 0.55	−0.17 (−0.25 to (−0.08)) 0.01 (−0.07 to 0.10) −0.32 (−0.54 to (−0.09)) −0.18 (−0.40 to (−0.05)) −0.33 (−0.74 to 0.90) −0.06 (−0.47 to 0.36) −0.75 (−1.49 to (−0.02)) −0.46 (−1.19 to 0.27)	.001* .782 .009* .116 .115 .770 .045* .201	10.138 .896 .958 .370	.007* .360 .344 .553	0.420 0.060 0.064 0.026
**Gait Scores**								
**Speed**	LSVT m‐LSVT	1.31 ± 0.30 1.40 ± 0.15	1.46 ± 0.15 1.50 ± 0.13	0.15 (0.06–0.24) 0.10 (0.01 to 0.19)	.004* .030*	.562	.466	0.039
**Cadence**	LSVT m‐LSVT	104.79 ± 9.78 111.27 ± 8.17	111.90 ± 11.30 115.94 ± 5.30	7.11 (1.10 to 13.12) 4.66 (−1.34 to 10.67)	.024* .118	.383	.546	0.027
**Step length (str length %)** Dominant side Nondominant Side	LSVT m‐LSVT LSVT m‐LSVT	48.20 ± 2.74 50.50 ± 1.22 50.00 ± 3.30 49.50 ± 1.22	49.86 ± 2.95 50.78 ± 2.12 51.80 ± 2.03 50.03 ± 0.87	1.66 (−0.30 to 3.62) 0.28 (−1.5 to 2.07) 1.80 (0.21–3.39) 0.53 (−0.92 to 1.99)	.088 .729 .031* .428	1.373 1.768	.271 .217	0.132 0.164
**Stance phase (cycle %)** Dominant side Nondominant side	LSVT m‐LSVT LSVT m‐LSVT	62.98 ± 1.10 58.86 ± 2.65 61.54 ± 1.11 59.07 ± 1.33	64.98 ± 3.41 58.63 ± 1.85 61.98 ± 2.76 60.17 ± 1.83	2.0 (−0.54 to 4.53) −0.23 (−2.55 to 2.08) .040 (−2.20 to 3.08) 1.10 (−1.31 to 3.51)	.108 .825 .715 .329	2.159 .174	.176 .686	0.194 0.019
**Swing phase (cycle %)** Dominant side Nondominant side	LSVT m‐LSVT LSVT m‐LSVT	36.94 ± 2.89 39.35 ± 4.39 36.38 ± 1.53 38.70 ± 2.24	40.46 ± 2.36 41.20 ± 5.26 38.08 ± 2.04 39.43 ± 1.77	3.52 (0.64–6.40) 1.85 (−0.78 to 4.48) 1.70 (0.18 to 3.21) 0.73 (−0.65 to 2.11)	.022* .146 .031* .260	.938 1.141	.358 .313	0.094 0.113
**Gait cycle symmetry index**	LSVT m‐LSVT	90.02 ± 4.72 93.60 ± 1.42	96.84 ± 1.43 95.11 ± 1.66	6.82 (4.08–9.56) 1.51 (−0.98 to 4.17)	<.001* .203	10.470	.010*	0.538

*Note*: *p < .05 for interaction (time × group) by analysis of variance (ANOVA).

Abbreviations: LOS, limits of stability; m‐CTSIB, the modified clinical test of sensory integration of balance; PS, postural stability; SD, standard deviation.

UPDRS‐III, BBS, and TUG results are presented in Table 3. The severity of motor symptoms and functional mobility improved in both groups (*p* < .05); these improvements were similar in both groups (*p* >  .05). The LSVT group showed significant improvements in functional balance (*p* < .05); however, there was no significant difference between the two groups in the posttest (*p* >  .05).

**TABLE 3:** Treatment effects on severity of motor symptoms, functional balance, and functional mobility for the Lee Silverman voice treatment (LSVT) group and modified LSVT (m‐LSVT) group at baseline and 4‐week follow‐up.

		Pretest Median (IQR)	Posttest Median (IQR)	Change post‐pre Median (IQR)	Within‐group *p*‐value	Between‐group *p*‐value
**UPDRS‐III**	LSVT m‐LSVT	21.5 (11.3–49.0) 21.50 (20.0–26.3)	15.0 (9.3–41.0) 18.5 (16.3–24.0)	−6.0 (−10.3 to (−2.0)) −3.0 (−5.5 to (−1.0))	.011* .017*	.712
**BBS**	LSVT m‐LSVT	55.0 (34.8–55.8) 54.5 (52.3–56.0)	56.0 (49.3–56.0) 56.0 (53.5–56.0)	1.0 (0.3–11.3) 0.5 (0.0–3.0)	.026* .098	.763
**TUG**	LSVT m‐LSVT	8.2 (6.9–22.1) 7.94 (7.0–9.0)	6.3 (5.9–11.3) 7.8 (6.0–8.5)	−1.3 (−7.4 to (−0.8)) −0.7 (−0.9 to (−0.6))	.011* .012*	.875

*Note*: *p < .05 for the within‐group by Wilcoxon signed rank test and the between‐group by the Mann–Whitney *U* test.

Abbreviations: BBS, Berg Balance Scale; TUG, timed up and go test; UPDRS‐III, unified Parkinson's disease rating scale‐motor score.

## 4 DISCUSSION

This is the first study to investigate the effects of a modified protocol (m‐LSVT group), with two one‐on‐one sessions per week and two sessions at home, unlike standard LSVT BIG training (four one‐on‐one sessions per week) in PD. This study showed significant group‐by‐time interactions in favor of the LSVT group on the eyes open‐firm surface score of sensory integration of balance and gait cycle symmetry index in PwPD. The within‐group changes demonstrated that both protocols significantly improved PS, gait speed, motor symptoms, and functional mobility. In addition, the stability limit, most scores of sensory integrations, functional balance, cadence, step length, swing phase, and gait cycle symmetry index results improved only in the LSVT group, indicating that the improvement in balance and gait was greater in this group.

Previous studies, mostly case studies (Fishel et al., 2020; Hirakawa et al., 2022; Iwai et al., 2021; Janssens et al., 2014; Kleppang & Jørgensen, 2020; Millage et al., 2017; Pascal et al., 2018), have shown that LSVT BIG training improves functional balance (Choi & Kim, 2022; Fishel et al., 2020; Hirakawa et al., 2022; Janssens et al., 2014; Kleppang & Jørgensen, 2020; Millage et al., 2017; Pascal et al., 2018) and PS (Iwai et al., 2021). The results of our study, which was a rater‐blinded randomized controlled trial, confirm the existing literature that LSVT BIG training improved balance. Additionally, it improved the limits of stability, and sensory integration of balance, which were other parameters of balance in this study. On the other hand, care was taken to ensure that the exercise intensity of the m‐LSVT group exercises was as intense as in the standard group, but the improvements were limited to PS in terms of within‐group change. LSVT BIG training emphasizes shaping the quality and movement amplitude through the use of modeling or tactile/visual cues (Fox et al., 2012). For this reason, PwPD practiced their exercises with a therapist during the sessions. Unlike the standard protocol, the m‐LSVT group performed the exercises one‐on‐one with a therapist only twice a week. We think that the less improvement of balance in the m‐LSVT group may be due to this.

Gait impairment is one of the main causes of disability in the daily life of PwPD (Shulman et al., 2008). In the LSVT BIG training, “BIG walking” is included as part of the hierarchy on a daily basis aimed at overcoming bradykinesia/hypokinesia during walking, and gait has an important place throughout the treatment (Fox et al., 2012). Ebersbach et al. (2010) found that LSVT BIG training improved motor performance more than Nordic walking exercise and unassisted home exercise. Schaible et al. (2021) found that the LSVT BIG training and an individualized intense exercise program were more effective than the standard rehabilitation program in terms of walking speed and step length. They also found that all three exercise programs were effective in improving motor symptoms. Additionally, a lot of case studies found that LSVT BIG training improved functional mobility (Fishel et al., 2020; Flood et al., 2020; Hirakawa et al., 2022; Janssens et al., 2014), gait speed (Flood et al., 2020; Kleppang & Jørgensen, 2020; Millage et al., 2017), stride length (Flood et al., 2020), and motor symptoms (Fırat et al., 2023; Hirakawa et al., 2022; Iwai et al., 2021; Janssens et al., 2014; McDonnell et al., 2018; Millage et al., 2017; Ueno et al., 2017). Matsuno et al. (2023) reported that LSVT BIG training improved motor symptoms, gait speed, and step length, but there was no improvement in stride duration, cadence, stance phase, and swing phase. Our results are consistent with previous studies that LSVT BIG training improved functional mobility, gait speed, nondominant side step length, swing phase, and gait cycle symmetry index, and motor symptoms. In another study, Ebersbach et al. (2015) compared the standard LSVT BIG protocol with a short LSVT BIG protocol (five times per week for 2 weeks at the clinic). The results showed that although motor performance improved equally in both groups, standard LSVT BIG was more effective in obtaining the patient‐perceived benefit. LSVT LOUD is a standard speech therapy protocol with the same training principles and intensity as LSVT BIG training (Fox et al., 2012). A review found results supporting that the online modified version of LSVT LOUD is not inferior to the standard protocol (Herd et al., 2012). In this study, we compared the standard LSVT BIG protocol (four times per week for 4 weeks at the clinic) with the modified version (twice a week for 4 weeks at the clinic and twice a week for 4 weeks at home). Although functional mobility, motor symptoms, and gait speed improved in both groups, there was no interaction between the groups (except gait cycle symmetry index). As a result, we demonstrate that the m‐LSVT BIG protocol could improve gait parameters, functional mobility, and motor symptoms in PwPD.

This study had some limitations. First, treatment programs required in‐clinic exercise two to four times a week. PwPD come from many cities of the country to the center, where patients’ eligibility criteria are evaluated. Because most of the excluded patients came from outside the city, they could not participate in this program, which required regular participation. In addition, although individuals in the modified group came to the clinic less frequently, one patient was unable to continue treatment. This was because he had difficulty reaching treatment alone and did not have a caregiver or family member who could help with transportation. For these reasons, telerehabilitation option may be considered in further studies. Another important exclusion criterion was to exclude all participants with advanced PD. Therefore, studies are needed in patients with advanced stages of the disease. All patients who completed the program in both groups participated in the sessions with great interest. One patient (standard LSVT group) even started coming to the clinic alone during his last sessions and was very pleased with this development. Another limitation of our study was that we did not evaluate the patient's satisfaction levels. We recommend that the satisfaction level be questioned in future studies to better understand the differences between the two groups. Third, there was no long‐term follow‐up. LSVT BIG training aims at the acquisition of motor skills (Fox et al., 2012), so long‐term follow‐up may provide a better understanding of the effects of the modified protocol. Finally, although seemingly adequately powered, the sample size in this study may still limit the generalizability of the study results. We recommend that future studies be conducted by including more patients.

## 5 CONCLUSION

This study showed the superiority of the standard protocol over the modified protocol in terms of sensory integration and gait cycle symmetry. However, motor symptoms, PS, functional mobility, and gait speed improved similarly in both groups. These results suggested the beneficial effects of the m‐LSVT BIG protocol on balance and gait in PwPD as well as the superiority of the standard LSVT BIG protocol. Therefore, the m‐LSVT BIG program may be an effective intervention method, especially for patients who have difficulty adapting to the standard protocol.

## AUTHOR CONTRIBUTIONS

**Sefa Eldemir**: Methodology; writing—original draft; supervision; formal analysis; conceptualization; writing—review and editing; visualization; investigation. **Kader Eldemir**: Writing—original draft; methodology; visualization; writing—review and editing. **Fettah Saygili**: Writing—review and editing; visualization. **Cagla Ozkul**: Writing—review and editing; methodology; visualization. **Rezzak Yilmaz and Muhittin Cenk Akbostancı**: Writing—review and editing; resources; supervision. **Arzu Guclu‐Gunduz**: Supervision; writing—review and editing; writing—original draft; methodology.

### PEER REVIEW

The peer review history for this article is available at https://publons.com/publon/10.1002/brb3.3458.

## Data Availability

Data are available on request from the authors.
